# Modeling road accident fatalities with underdispersion and zero-inflated counts

**DOI:** 10.1371/journal.pone.0269022

**Published:** 2022-11-17

**Authors:** Teerawat Simmachan, Noppachai Wongsai, Sangdao Wongsai, Rattana Lerdsuwansri

**Affiliations:** 1 Faculty of Science and Technology, Department of Mathematics and Statistics, Thammasat University, Pathum Thani, Thailand; 2 Thammasat University Research Unit in Data Learning, Thammasat University, Pathum Thani, Thailand; Southeast University, CHINA

## Abstract

In 2013, Thailand was ranked second in the world in road accident fatalities (RAFs), with 36.2 per 100,000 people. During the Songkran festival, which takes place during the traditional Thai New Year in April, the number of road traffic accidents (RTAs) and RAFs are markedly higher than on regular days, but few studies have investigated this issue as an effect of festivity. This study investigated the factors that contribute to RAFs using various count regression models. Data on 20,229 accidents in 2015 were collected from the Department of Disaster Prevention and Mitigation in Thailand. The Poisson and Conway–Maxwell–Poisson (CMP) distributions, and their zero-Inflated (ZI) versions were applied to fit the data. The results showed that RAFs in Thailand follow a count distribution with underdispersion and excessive zeros, which is rare. The ZICMP model marginally outperformed the CMP model, suggesting that having many zeros does not necessarily mean that the ZI model is required. The model choice depends on the question of interest, and a separate set of predictors highlights the distinct aspects of the data. Using ZICMP, road, weather, and environmental factors affected the differences in RAFs among all accidents, whereas month distinguished actual non-fatal accidents and crashes with or without deaths. As expected, actual non-fatal accidents were 2.37 times higher in April than in January. Using CMP, these variables were significant predictors of zeros and frequent deaths in each accident. The RAF average was surprisingly higher in other months than in January, except for April, which was unexpectedly lower. Thai authorities have invested considerable effort and resources to improve road safety during festival weeks to no avail. However, our study results indicate that people’s risk perceptions and public awareness of RAFs are misleading. Therefore, nationwide road safety should instead be advocated by the authorities to raise society’s awareness of everyday personal safety and the safety of others.

## Introduction

In 2013, Thailand ranked second, after Libya, in road accident fatalities (RAFs) in a survey of 180 countries worldwide, with an estimated 36.2 deaths in road traffic accidents (RTAs) per 100,000 people [1]. In 2016, Thailand had an estimated rate of 32.7 RAFs and thus ranked eighth among 175 countries and was first in Southeast Asia [2]. Using the RAF prediction model as a function of registered vehicles per capita, the estimate was 30.68 deaths per 100,000 population in 2020 [3]. According to a 2020 report by the World Health Organization (WHO), these estimates of over 30 deaths per 100,000 people have remained virtually constant over the past decade, serving as evidence against the Thai government’s target for 2021 of 18 deaths as declared in the Road Safety Master Plan (2018–2021) [4]. Thailand seems to be impervious, to the target of 10 deaths in the Decade of Action for Road Safety (2001–2020) or the United Nations Sustainable Development Goal of 3 and its associated target of 3.6 in reducing the number of global deaths and injuries from RTAs by 50% by 2020. This is also happening in other countries and thus the declaration of the second Decade of Action for Road Safety 2021–2030 ensues, with the goal of reducing RAFs by at least 50% by 2030 [5].

Problems with the quality of road safety databases are critical in Thailand. The country’s RAFs are particularly vulnerable to underreporting. In 2013, 14,056 deaths (20.98 deaths rate) were reported by the Office of the Permanent Secretary of the Ministry of Public Health. This was much lower than the estimated 24,237 deaths (36.2) according to the WHO report. In response, the government emphasized its agenda on road safety issues. The lack of data integration was traced and completed in 2016. The data were managed and collected from three public sectors: (1) the Injury Surveillance System of the Ministry of Public Health (death registration confirmed with medical certification of the cause of death from hospitals); (2) the Police Information System by the Royal Thai Police, Ministry of Interior; and (3) the E-Claim System by Road Accident Victims Protection Company Limited (all claim petitions and compensations related to RTAs nationwide). With these collaborative efforts, the estimates by the WHO in 2016 (22,491 deaths) and the country’s report (21,745 deaths) were nearly synchronized.

In 2018, the Injury Data Collaboration Center (IDCC), responsible for the management and maintenance of a national database on road safety, was established [6]. The RAF report for 2011–2020 over 76 provinces and the capital city of Bangkok can be accessed via the IDCC website, https://dip.ddc.moph.go.th/new/. RAFs reported in Thailand are defined for an unlimited period following a crash, not immediately after a crash, or within 30 days after a traffic accident [7].

**Fig 1:**
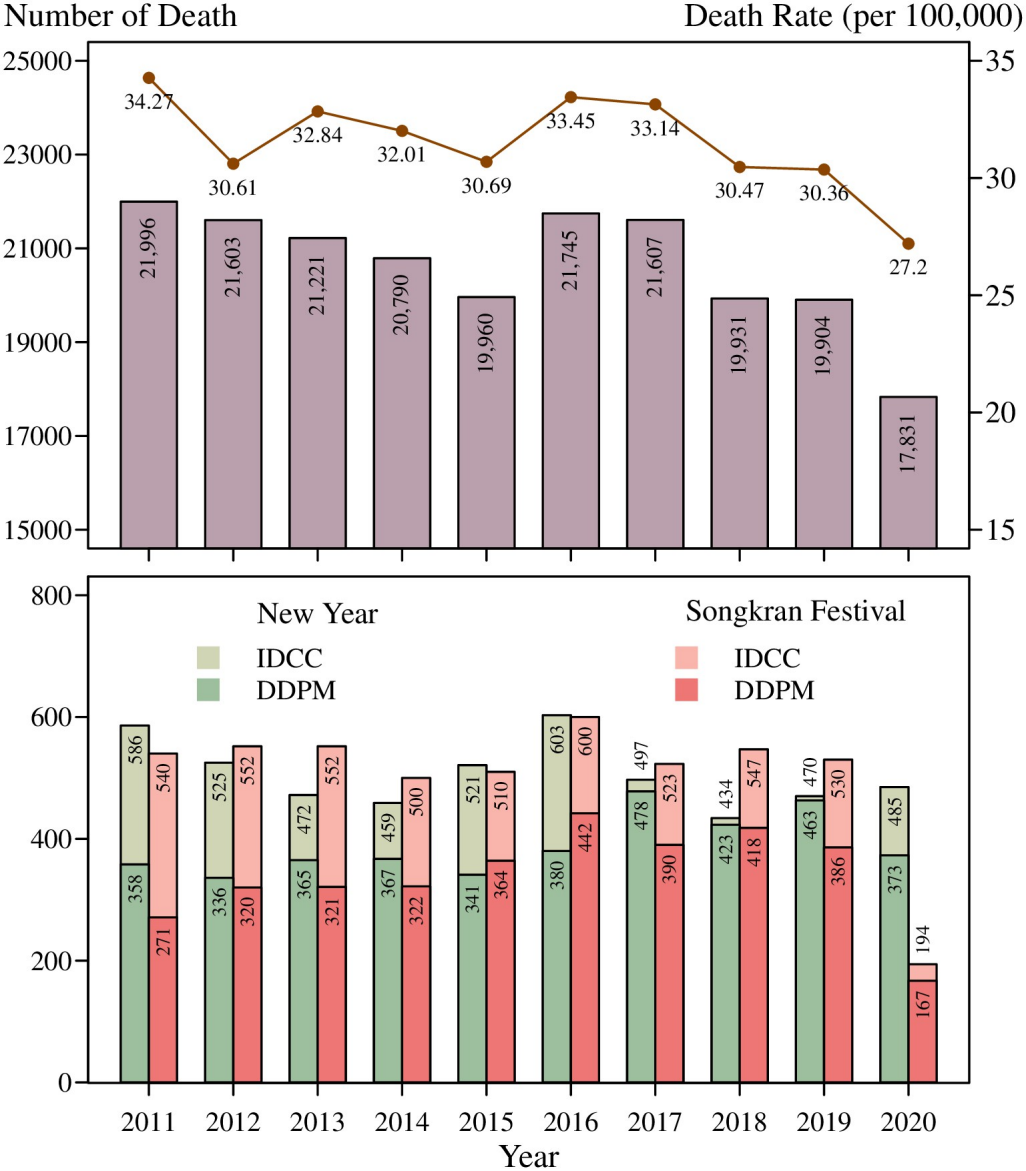
Number of deaths and death rate (per 100,000 population) on roads in Thailand for the 2011–2020 decade: (above) overall and (below) during the 7-day New Year Celebration and 7-day Songkran Festival, based on IDCC and Department of Disaster Prevention and Mitigation (DDPM) reports.

The focus is not only on RAFs over the years, but also on the numbers throughout the year, which is critical, particularly during the Songkran festival and New Year holidays. Fig 1 shows the RAFs over the past decade (2011–2020). The RAFs for 2011–2018 involved more than 20,000 deaths per year and over 30 deaths per 100,000 inhabitants, representing approximately 58 deaths per day. During the long seven-day holiday, the number of RAFs rose to approximately 77 and 73 deaths per day during the Songkran festival and New Year holidays, respectively. In addition, real-time monitoring of RAF data over the seven-day deadly festivals shows that more than 80% of victims died after a crash [8]. The lockdown policy for the surveillance, detection, and control of the COVID-19 epidemic in 2019–2020 appears to have saved lives on the road. The lower number of cars on the roads and reduced traffic volume have been proven to reduce RAFs. Other factors affecting the number of RTAs, RAFs, and road accident injuries (RAIs) must be addressed to improve preventive measures and overcome problems in the long term.

Previous studies in Thailand have focused on human behavior and demographics rather than road and environmental factors to explain road safety incidences. Over the past two decades, RTAs, RAFs, and RAIs have been dominated by motorcycles, young people, drunk driving, and not wearing a helmet [4,9–13], suggesting the failure of road safety campaigns and law enforcement. Few studies have considered the road and environmental factors. The number of RTAs increased significantly with a higher volume of rainfall in the southern and northern provinces during the period 2012–2018 [14]. These figures on Thai highways for 2011–2017 were affected by the length of the segment and average annual traffic volume [15]. Road type, road section, and month are crucial factors impacting the number of injuries and fatalities on roads [16,17].

Count regression models for road safety have been reported elsewhere (Table 1). Several studies have estimated the frequency of RTAs, RAFs, or RAIs using traditional Poisson regression, negative binomial (NB) regression, or Conway–Maxwell–Poisson (CMP) regression. The model chosen depends on the assumption of equidispersion. When it is held, standard Poisson regression is simply applied [18], and a further adjustment with a spatial correlation has recently been presented [19]. In other cases, NB is commonly used to handle the overdispersion problem [20–23] and other versions such as NB-1, NB-2, and Bayesian NB have been developed [24–27]. Alternatively, CMP has recently been adopted to overcome overdispersion [28,29] and underdispersion [30], although only a few published studies have addressed the latter.

**Table 1:** Previous studies on road safety using count regression and zero-inflated models. For model comparison, if any, the model that performed best is presented in boldface.

Study	Outcome variable	Model
**Equidispersion**		
Michener and Tighe [18]	Crash frequency	Poisson
Hezaveh **et al**. [19]	Crash frequency	Poisson and **Geographically Weighted Poisson**
**Underdispersion**		
Lord et al. [30]	Crash frequency	Poisson, **CMP** and Gamma
**Overdispersion**		
Chen et al. [20], Quistberg **et al**. [23]	Number of fatalities	NB
Obinguar and Iryo-Asano [21]	Pedestrian crash frequency	NB
Das [22]	Crash frequency	NB
Khattak **et al**. [24]	Crash frequency	NB-1, NB-2, **NB-P** and generalized Poisson
Kamla et al. [25]	Counts of harsh braking incidents	**NB Random-parameters model** and NB Fixed-parameters model
Safaei et al. [26]	Number of fatal accidents	random effect NB
Cai et al. [27]	Crash frequency, Number of injuries, Number of fatalities	Bayesian NB
Lord et al. [28]	Crash frequency	CMP
Khan et al. [29]	Crash frequency	CMP with gamma random effects
**Overdispersion with excessive zeros**		
Mansfield et al. [31]	Number of pedestrian fatalities	NB, ZINB and **ZINB mixed model**
Raihan et al. [32]	Crash frequency	ZINB
Wan et al. [33]	Crash frequency	P, **NB**, ZIP and ZINB
Mathew and Benekohal [34]	Crash frequency	ZINB with Empirical Bayes adjustments
Cloutier and Lachapelle [35]	Number of fatal accidents	ZINB

Both dispersion and excessive zero counts are concerns in count data analyses. Searching in the Elsevier Science Direct database for the period 2018–2022 using the keywords: “excessive zeros and road fatality, road accident or road injury,” 63 studies were identified that dealt with many zero counts and overdispersion problems using zero-inflated models (see for example [31–35]). To the best of our knowledge, no study has analyzed accident data with underdispersion and excessive zeros. Moreover, classifying different injury severity levels is also of interest in road accident studies.

Logistic regression models have been seen in the literature [36–38], along with a range of machine learning techniques, such as regression trees, random trees, random forests, support vector machines, k-nearest neighbors, and deep neural networks [39–44]. In addition to count data and categorical data, there is considerable interest in the traffic congestion rate. To account for unobserved heterogeneity across observations, the models under Bayesian frameworks have been proposed in studies of pedestrian safety and signalized intersections [45,46]. To deal with sophisticated models, Bayesian methods have been employed to estimate model parameters, such as studies of correlations and heterogeneity in crash rates [47] and for cyclist safety [48].

However, the predictive models for death and/or injury are limited to Thailand’s road safety. The present study fills in this gap in research by using count regression models to investigate the distribution of RAFs in Thailand and their potential association with the road and environment at the accident location.

The remainder of this paper is organized as follows. In the Methods section, a detailed description of the data source is given, and count regression models and test statistics for dispersion, excessive zeros, and goodness of model fitting are discussed. Model selection with the best performance in identifying key factors is provided in the Results section. The Discussion section interprets the results and compares them with previously reported results. The Conclusion section summarizes the overall study findings in terms of the differences between the death counts and the extra zero death counts, as well as the limitations of the study.

## Methods

### Data

Data were collected from the DDPM, Ministry of Interior, for 2015, which was the most recent year for which data are available at the time of this study. A total of 76 disaster prevention and mitigation provincial offices are located across Thailand to handle disaster management at the local level. Each office handles data collection and analysis and reports on disaster damage and loss due to natural and man-made disasters, including RTAs.

In 2015, DDPM reported 25,586 RTAs from emergency aid requests for crash accident victims at the provincial and district levels. Complete data were available for 20,229 cases. The unit of analysis here is the individual RTA in a year of study; the dependent variable is the number of human deaths, and the independent variables are the road characteristics (class, surface, and section), weather conditions, environmental conditions, and month of the year. Covariate variables and their specifics are detailed in Table 2. The national database on road safety supplies public information on the number of RTAs, RAFs, types of vehicles, human demographics, and behaviors (which are beyond the scope of this study).

**Table 2:** Definition of independent variables.

Variable	Description
**Roadway class**	
1: National highway	Road networks that are under the control of the Department of Highways, Ministry of Transport.
2: Rural highway	Road networks that are under the control of the Department of Rural Roads, Ministry of Transport.
3: Urban road	Road networks that are under the control of the local municipality, Ministry of Interior.
4: Local street	Road networks that are under the control of the local subdistrict administration organization, Ministry of Interior.
**Road surface**	
1: Dry	Road skin is in a dry condition that exposes its original surface material.
2: Wet	Road skin is in a wet condition due to the rainstorm or the remaining water after the rainfall. Substantial water on the road causes slippery surfaces and hydroplaning.
**Road section**	
1: Straight	Road section is in a straight line, not horizontal, curved, or crooked, continuing in the same direction without deviating.
2: Curve	Road section is in both horizontal and vertical curves or bends directions.
3: Crossing and others	Other environmental properties of the road section include junctions, pedestrian crossings, and roadwork obstacles.
**Weather condition**	
1: Clear	A condition in which a road accident occurs in sunny weather, not cloudy, rainy, foggy, smoky, or dusty conditions that reduce daylight or visibility.
2: Fog	A condition in which a road accident occurs in foggy weather, which appreciably reduces visibility. It may include smoke from roadside fire and dust from roadwork and nearby construction.
3: Rain	A condition in which a road accident occurs in rainy weather, which appreciably reduces visibility and causes wet road surfaces.
**Light condition**	
1: Day	A condition in which a road accident occurs in daylight.
2: Night with light	A condition in which a road accident occurs in the dusk or darkness with artificial light (from streetlights).
3: Night without light	A condition in which a road accident occurs in the dusk or darkness without artificial light (from streetlights).
**Month**	
1: Jan–12: Dec	The month of year in which the accident occurred.

The road networks in Thailand are classified according to four governing authorities. The national highways are under the control of the Department of Highways. Four levels of national highways are differentiated by the number of digits in their name. The first-level networks connect Bangkok to four regions in Thailand (North, South, Northeast, and Central). The second-level networks connect the first-level networks to several significant provinces over long distances and spread out. The third-level networks connect the first and second-level networks to key locations in many provinces, including the long-distance road parallel to the border and attaching the higher-level networks. Finally, highways at the fourth level link other networks to provinces, districts, or important strategic locations in the provinces.

All first-level highways are either 4-lane, 6-lane, or 8-lane divided roads supporting high traffic density. Most second and third-level roads are mixed between 4-lane divided and undivided roads. The four-level highways are 2-lane undivided roads. Rural highways are constructed and maintained by the Department of Rural Roads. This class of roads links all levels of national highways to districts, sub-districts, and villages. Likewise, it connects places within districts and sub-districts. Urban roads comprise the networks within the major city areas under the authority of the local municipality. These networks typically comprise high-density roads with many junctions, traffic signals, and crossings. Local streets are narrow concrete-surface roads, with some being gravel in villages, built and maintained by the local subdistrict administration organization.

### Models

In this study, we applied standard Poisson, CMP, zero-inflated Poisson (ZIP), and zero-inflated CMP (ZICMP) regressions to model the number of fatalities in accidents for the year 2015 in relation to road characteristics, weather conditions, environmental conditions, and month of the year.

### Poisson regression

The Poisson distribution is appropriate for the dependent variable *Y*, taking nonnegative integer values 0, 1, 2, …. This can be used to model the number of occurrences of an event. In this study, *Y* represents the number of deaths per accident. The probability mass function (p.m.f.), mean, and variance of the Poisson distribution are listed in Table 3. Poisson regression is limited by the assumption imposed on its variance. The expected death counts *λ*_*i*_ at the accident *i* (*i* = 1, 2, …, *n*) are calculated using $\lambda_{i}=\text{exp}({\text{X}}_{i}^{\prime }\beta )$ to ensure *λ*_*i*_ > 0. ${\text{X}}_{i}^{\prime }$ is the vector of covariates and **β** is the vector of estimable coefficients. Given the p.m.f., link function, and the assumption of independent observations, the log-likelihood function for observation *i* is given as

$$\text{ln}\;L_{i}(\beta )=-\text{exp}({\text{X}}_{i}^{\prime }\beta )+y_{i}{\text{X}}_{i}^{\prime }\beta -\text{ln}\;y_{i}!$$ (1)

**Table 3:** Probability mass function (p.m.f.), mean, and variance for count distributions.

Model	p.m.f.	*E*(*Y*)	Var(*Y*)
**Poisson**	$\text{Pr}(Y=y)=\frac{e^{-\lambda }\lambda^{y}}{y!},\;y=0,1,2,\ldots ,\lambda >0$	*λ*	*λ*
**ZIP**	$\begin{array}{l} \text{Pr}(Y=0)=\pi +\left(1-\pi \right)e^{-\lambda },\;\lambda >0,\;0\leq \pi \leq 1,\\ \text{Pr}(Y=y)=\left(1-\pi \right)\frac{e^{-\lambda }\lambda^{y}}{y!},\;y=1,2,3,\ldots \end{array}$	(1 − *π*) *λ*	*λ* (1 − *π*) (1 + *πλ*)
**CMP**	$\text{Pr}(Y=y)=\frac{\lambda^{y}}{{\left(y!\right)}^{\nu }Z\left(\lambda ,\nu \right)},\;y=0,1,2,\ldots \;,$ $Z\left(\lambda ,\nu \right)=\sum_{j=0}^{\infty }\frac{\lambda^{j}}{{\left(j!\right)}^{\nu }},\;\nu \geq 0,\;\lambda >0$	$\lambda \frac{\partial \;\text{log}\;Z\left(\lambda ,\nu \right)}{\partial \lambda }\approx \lambda^{1/\nu }-\frac{\nu -1}{2\nu }$	$\lambda \frac{\partial E(Y)}{\partial \;\text{log}\;\lambda }\approx \frac{1}{\nu }\lambda^{1/\nu }$
**ZICMP**	$\text{Pr}(Y=0)=\pi +(1-\pi )\frac{1}{Z\left(\lambda ,\nu \right)},\;0\leq \pi \leq 1,$ $\text{Pr}(Y=y)=(1-\pi )\frac{\lambda^{y}}{{\left(y!\right)}^{\nu }Z\left(\lambda ,\nu \right)},\;y=1,2,3,\ldots \;,$ $Z\left(\lambda ,\nu \right)=\sum_{j=0}^{\infty }\frac{\lambda^{j}}{{\left(j!\right)}^{\nu }},\;\nu \geq 0,\;\lambda >0$	$(1-\pi )\frac{1}{Z\left(\lambda ,\nu \right)}\sum_{j=0}^{\infty }\frac{j\lambda^{j}}{{\left(j!\right)}^{\nu }}$	$(1-\pi )\frac{1}{Z\left(\lambda ,\nu \right)}\sum_{j=0}^{\infty }\frac{j^{2}\lambda^{j}}{{\left(j!\right)}^{\nu }}-E{\left(Y\right)}^{2}$

* *Z* (*λ*, *ν*) is a normalizing constant.

Summing over *n* observations, the log-likelihood function is

$$\text{ln}\;L(\beta )=\sum_{i=1}^{n}\left(-\text{exp}({\text{X}}_{i}^{\prime }\beta )+y_{i}{\text{X}}_{i}^{\prime }\beta -\text{ln}\;y_{i}!\right)$$ (2)

### Conway–Maxwell–Poisson regression

The CMP distribution is a generalization of the Poisson distribution that serves to model both underdispersed and overdispersed data. This distribution was originally proposed by Conway et al. [49] and subsequently implemented by Shimueli et al. [50] and Sellers et al. [51]. The p.m.f., mean, and variance are summarized in Table 3. CMP regression overcomes the restrictive assumption of Poisson regression by defining an additional dispersion parameter, $\nu_{i}=\text{exp}({\text{S}}_{i}^{\prime }\delta )$. ${\text{S}}_{i}^{\prime }$ is the vector of covariates and **δ** is the vector of estimable coefficients. The likelihood function for observation *i* has the form

$$\begin{array}{l} L(\lambda ,\nu )=\frac{\prod_{i=1}^{n}\lambda^{y_{i}}}{{\left(\prod_{i=1}^{n}y_{i}!\right)}^{\nu }Z{\left(\lambda ,\nu \right)}^{n}} \end{array}$$ (3)

The log-likelihood function can be written as

$$\text{ln}(L)=\sum_{i=1}^{n}\left(y_{i}\;\text{ln}(\lambda )-\nu \;\text{ln}(y_{i}!)\right)-n\;\text{ln}\left(Z(\lambda ,\nu )\right)$$ (4)

### Zero-inflated Poisson regression

The ZIP distribution was used to model count data with an excess of zero counts. It is assumed that with probability *π*, the only possible observation is zero, and with probability 1 − *π*, a Poisson (*λ*) random variable is observed. In our case, the former represents the proportion of actual non-fatal accidents. It is defined by always-zero death counts. The latter represents the proportion of crashes with or without fatalities, that are natural count data including zeros. A crash with at least one death is a fatal accident. A crash without fatalities is a common non-fatal accident. It is defined by not-always-zero death counts. Our zeros are a mixture of actual and common non-fatal accidents. The ZIP distribution has the following form:

$$f_{ZIP}(y_{i};\lambda_{i},\pi_{i})=\pi_{i}Poisson(y_{i};0)+\pi_{i}Poisson(y_{i};\lambda_{i})$$ (5)

The resulting p.m.f., mean, and variance are summarized in Table 3.

The ZIP model can be generalized by a regression model that allows both the Poisson parameter *λ* and the weight parameter *π* to vary. The model has two parts: the Poisson count model predicting zeros and non-zero counts (count component of the model), and the logit model predicting excess zeros, which is the so-called always zeros (zero component of the model). $W_{i}{}^{\prime }$ is the vector of covariates and **γ** is the vector of estimable coefficients. The logit link is defined as follows:

$$\text{logit}(\pi_{i})=\text{ln}\left(\frac{\pi_{i}}{1-\pi_{i}}\right)={\text{W}}_{i}^{\prime }\gamma \Rightarrow \pi_{i}=\frac{\text{exp}({\text{W}}_{i}^{\prime }\gamma )}{1+\text{exp}({\text{W}}_{i}^{\prime }\gamma )}$$ (6)

If *π* is not a function of *λ*, then the log-likelihood function for the ZIP regression is

$$\begin{array}{ll} \text{ln}\;L(\gamma_{i},\beta_{i}) & =\sum_{y_{i}=0}^{}\text{ln}\left(\text{exp}({\text{W}}_{i}^{\prime }\gamma )+\text{exp}\left(\text{exp}(-{\text{X}}_{i}^{\prime }\beta )\right)\right)+\sum_{y_{i}>0}^{}\left(y_{i}{\text{X}}_{i}^{\prime }\beta -\text{exp}(-{\text{X}}_{i}^{\prime }\beta )\right)\\ & -\sum_{i=1}^{n}\text{ln}\left(1+\text{exp}({\text{W}}_{i}^{\prime }\gamma )\right)-\sum_{y_{i}>0}^{}\left(y_{i}!\right) \end{array}$$ (7)

### Zero-inflated Conway–Maxwell–Poisson regression

A suggested model for underdispersed or overdispersed count data with excessive zeros is the ZICMP distribution proposed by Sellers et al. [52]. This generalization of the ZIP distribution is a mixture of a degenerate distribution at zero with probability *π* and a *CMP*(*λ*, *ν*) distribution with probability 1 − *π*. The resulting p.m.f., mean, and variance are summarized in Table 3. The ZICMP regression allows both the CMP parameter (*λ*, *ν*) and weight parameter *π*. It has two parts: the CMP count model and the logit model. The log-likelihood function for the ZICMP model is given by

$$\begin{array}{ll} \text{ln}(L) & =\sum_{y_{i}=0}\text{ln}\left\{\pi_{i}+\left[1-\pi_{i}\right]\frac{1}{Z(\lambda_{i},\nu_{i})}\right\}\\ & +\sum_{y_{i}>0}\left\{\text{ln}\left[1-\pi_{i}\right]-\text{ln}\left(Z(\lambda_{i},\nu_{i})\right)+\left(y_{i}\;\text{ln}(\lambda_{i})-\nu_{i}\;\text{ln}(y_{i}!)\right)\right\} \end{array}$$ (8)

The maximum likelihood method was used to estimate the coefficients of all four regression models used in this study.

### Dispersion test

The question arises as to whether there is any evidence of dispersion. First, in exploratory data analysis, when the mean of death counts is greater (less) than its variance, an underdispersion (overdispersion) is observed. In addition, the likelihood ratio test (LRT) can be performed to test the dispersion in the CMP regression. According to the relationship between the mean and variance of the CMP distribution

$$Var(Y)=\lambda \frac{\partial }{\partial \lambda }E(Y)\approx \lambda \frac{\partial }{\partial \lambda }\left(\lambda^{1/\nu }-\frac{\nu -1}{2\nu }\right)=\frac{1}{\nu }\lambda^{1/\nu }\approx \frac{1}{\nu }E(Y)$$ (9)

such that $\frac{Var(Y)}{E(Y)}\approx \frac{1}{\nu }$. This yields overdispersion and underdispersion for *ν* < 1 and *ν* > 1, respectively. In particular, when *ν* = 1, the CMP model collapses into the Poisson model. Hence, the test of *H*_0_: *ν* = 1 versus *H*_1_: *ν* ≠ 1 can be interpreted as testing whether the Poisson model is preferred over the CMP model. H_1_ does not specify the direction of data dispersion. In this case, the LRT statistic is given by

$$-2\;\text{log}\;\Lambda =-2\left[\text{log}\;L({\hat{\beta }}^{\left(0\right)},\hat{\nu }=1)-\text{log}\;L(\hat{\beta },\hat{\nu })\right]$$ (10)

where ${\hat{\beta }}^{\left(0\right)}$ is the maximum likelihood estimates (MLEs) obtained under H_0_ (the Poisson estimates); $\hat{\beta }$ and $\hat{\nu }$ are the maximum likelihood estimates under H_1_ (the CMP model). The LRT statistic is asymptotically distributed as a chi-squared distribution with one degree of freedom [52].

Similarly, ZICMP can be reduced to ZIP if *ν* = 1. The ratio of two log-likelihood functions of ZIP and ZICMP can be used in the following form $\frac{L(\hat{\beta },\hat{\nu }=1,\hat{\gamma })}{L(\hat{\beta },\hat{\nu },\hat{\gamma })}$. The LRT statistic is specified as

$$-2\left[\text{log}\;L(\hat{\beta },\hat{\nu }=1,\hat{\gamma })-\text{log}\;L(\hat{\beta },\hat{\nu },\hat{\gamma })\right]\sim \chi_{1}^{2}$$ (11)

where $\text{log}\;L(\hat{\beta },\hat{\nu }=1,\hat{\gamma })$ and $\text{log}\;L(\hat{\beta },\hat{\nu },\hat{\gamma })$ are the maximized log-likelihood under the ZIP and ZICMP regression models, respectively.

### Zero-inflation test

The score test for zero-inflation in a Poisson distribution is to determine whether an extra proportion of zeros (always zeros part) is added to the common count part of the discrete Poisson distribution. Under the null hypothesis (*H*_0_: *π* = 0), the score statistic has an asymptotic chi-squared distribution with one degree of freedom [53]. The score statistic is defined as follows:

$$S=\frac{{\left(n_{0}-np_{0}\right)}^{2}}{np_{0}\left(1-p_{0}\right)-n\bar{y}p_{0}^{2}}$$ (12)

where *n* is the total number of observations; *n*_0_ is the number of zeros in the data; $\bar{y}$ is the mean of the death counts (the estimate of the Poisson parameter under the null hypothesis); and $p_{0}=\text{exp}(-\bar{y})$.

Typically, CMP is a flexible two-parameter distribution for overdispersed and underdispersed count data. The question of addressing the excessive number of zero counts under CMP is of interest, leading to the ZICMP model. To test the CMP model against the ZICMP alternative, the LRT test is determined by $-2\left[\text{log}\;L(\hat{\beta },\hat{\nu },\hat{\gamma }=0)-\text{log}\;L(\hat{\beta },\hat{\nu },\hat{\gamma })\right]\sim \chi_{1}^{2}$, where $\text{log}\;L(\hat{\beta },\hat{\nu },\hat{\gamma }=0)$ and $\text{log}\;L(\hat{\beta },\hat{\nu },\hat{\gamma })$ are log-likelihood values associated with CMP MLEs and ZICMP MLEs, respectively.

### Goodness of fit and model comparison

To assess model adequacy, the generalized Pearson χ2 statistic is used, and it is computed as follows:

$$\chi^{2}=\sum_{i=1}^{n}\frac{{\left(y_{i}-E(Y_{i})\right)}^{2}}{E(Y_{i})}$$ (13)

The numerator is the squared difference between the observed death count and the expected value of the death count, and the denominator is the expected value of the death count. In large samples, the distribution of this statistic is approximately chi-squared with n − k degrees of freedom, where n is the total number of observations, and k is the number of estimated parameters, including the intercept. A small value of the statistic supports the decision not to reject the null hypothesis. If the null hypothesis cannot be rejected, then the model is adequate.

The assessment criteria used for model comparisons are often based on several likelihood measures. Two of the most extensively used measures are the log-likelihood value and Akaike Information Criterion (AIC). A model that provides a larger log-likelihood value is considered a better model. The AIC is defined as $AIC=-2\;\text{ln}\;L(\hat{\theta })+2k$, where $L(\hat{\theta })$ denotes the likelihood function of a given fitted regression model and $\hat{\theta }$ is a vector of the estimated parameters in the model. The smaller the AIC, the better the model.

### Software used

The results of this study were obtained using R 4.1.0 [54] with four main packages for count models: MASS, pscl, COMPoissonReg, and vcdExtra. The standard Poisson model within a generalized linear model framework was implemented in the R package MASS using the glm() function [55]. The zero-inflated extension of the Poisson distribution is provided by the zeroinfl() function in the pscl package [56]. Currently, implementation of the count model for mean, dispersion, and zero-inflation is available in the COMPoissonReg package using the glm.cmp() function [57]. The score test statistic is widely used to test the null hypothesis that the data do not characterize the extra zeros for the Poisson distribution. The score test [53] was implemented as the zero.test() function in R under the package vcdExtra [58].

## Results

### Descriptive statistics

**Fig 2:**
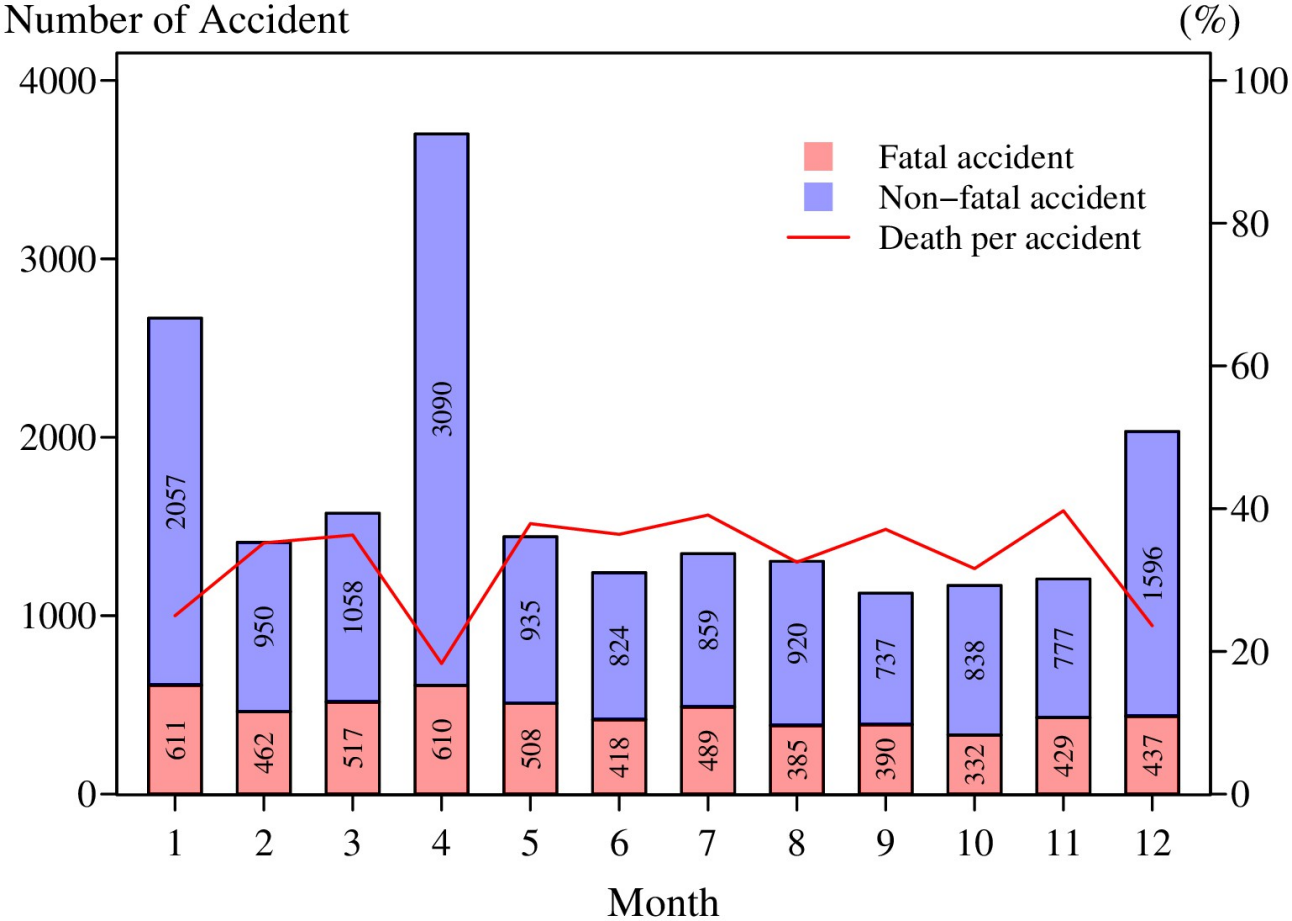
Statistics of the number of accidents and fatalities per accident by month in 2015.

The distribution of the death counts is summarized in Table 4 and presented in Fig 2. The number of deaths per accident in 2015 ranged from 0 to 6, with many accidents reporting 0 or 1 death per crash. The proportion of deaths was the highest for the zero count (73%) and the second highest for one count (25.79%). Fatal accidents accounted for approximately 27% of the total incidence (n = 20,229) and 6,109 deaths. 73% were non-fatal accidents, in which at least one person was injured, but no deaths occurred. The minimum number of fatal accidents was 332 (in October) and the maximum was 611 (in January). Non-fatal accidents were observed at least in September (737) and most in April (3,090).

**Table 4:** Variable description and the number of RTAs (percentage) in Thailand in 2015 (n = 20,229).

Variable	RTA (%)	Variable	RTA (%)
Fatality		Weather condition	
0: No person killed	14,641 (72.38)	1: Clear	18,797 (92.92)
1: One person killed	5,218 (25.79)	2: Fog	792 (3.92)
2: Two persons killed	285 (1.41)	3: Rain	640 (3.16)
3: Three persons killed	45 (0.23)	Light condition	
4: Four persons killed	23 (0.11)	1: Day	12,360 (61.10)
5: Five persons killed	8 (0.04)	2: Night with light	4,615 (22.81)
6: Six persons killed	9 (0.04)	3: Night without light	3,254 (16.09)
Roadway class		Month	
1: National highway	8,737 (43.19)	1: January	2,668 (13.19)
2: Rural highway	2,738 (13.54)	2: February	1,412 (6.98)
3: Urban Road	2,977 (14.72)	3: March	1,575 (7.79)
4: Local Street	5,777 (28.56)	4: April	3,700 (18.29)
Road surface		5: May	1,443 (7.13)
1: Dry	18,752 (92.70)	6: June	1,242 (6.14)
2: Wet	1,477 (7.30)	7: July	1,348 (6.66)
Road section		8: August	1,305 (6.45)
1: Straight	13,750 (67.97)	9: September	1,127 (5.57)
2: Curve	2,478 (12.25)	10: October	1,170 (5.78)
3: Crossing and others	4,001 (19.78)	11: November	1,206 (5.96)
		12: December	2,033 (10.05)

The monthly RTA averages were 465.7, 1,220.1, and 1,685.8, for fatal, non-fatal, and total accidents, respectively. The number of RTAs was highest in April, followed by January and December. In these months, important festivals are celebrated in Thailand, with long holiday periods of more than five days. However, the fatalities per accident per month were lower in these festival months than in other months. The highest proportion (0.396) was observed in November and the lowest (0.165) in April. A greater number of non-fatal injuries was observed during celebrations. This plays a crucial role in excessive zero participation in the data.

Six explanatory variables were used to estimate RAFs (Table 4). Many accidents occurred on national (43%) and rural highways (14%). These highways are constructed and maintained by the central government. The remaining accidents occurred on urban roads (15%) and local streets (28%). They are built and maintained by local government administrations. The national highway in Thailand covers 52,189 km, accounting for 7.5% of the road network in the country, the rural highway covers 49,123 km (7.0%), and the local government administration roads and streets cover 597,667 km (85.5%). Interestingly, although national highway coverage is 11.4 times lower than the urban road and the local street network, there seems to be an equal chance of RTAs (43%) occurring on both networks. Unfortunately, data on vehicle types and traffic volumes for the various types of roads are not available. Consequently, we cannot report an average of vehicles per day on the roads and which vehicle type occurs the most.

The most common road geometry for which the accidents occurred was straight section roads (68%), followed by pedestrian crossings (20%), and then curves (12%). RTAs were two times as high on straight road sections compared to the crossings and curved sections combined. Dry surface roads were at elevated danger (93%), whereas roads with wet surfaces were associated with lower danger (7%). Most accidents occurred under clear weather conditions (93%). Driving on dry surface roads and under clear weather may increase the risk of fatal accidents because drivers are less cautious in these conditions.

In terms of light conditions, accidents were reported in descending order of daylight (61%), at night with light on (23%), and at night without light (16%). This suggests that the visibility of the driver is one of the main factors involved in the cause of RTAs. During festival months, New Year celebrations (December: 10.1% and January: 13.2%), and Songkran holidays (April: 18.3%) the frequency of accidents increased, resulting in a sizable number of fatalities and injuries.

### Fitting fatality count distributions

Table 5 presents the results of fitting the death count data using the four probability models. When fitting the data to the Poisson distribution, the average number of fatalities per road accident was 0.302 (exp(-1.1974)). The estimated dispersion parameter in the Poisson distribution is less than one, indicating count data with underdispersion. This was improved by fitting the CMP model to the data. The estimated dispersion parameter was 1.55 (exp(0.4384)), denoting underdispersion relative to the Poisson model). The LRT statistic for dispersion is 85.87 and is clearly significant with one degree of freedom and p-value < 0.0001 (refer to Eq (10)). The expected number of deaths increased to 0.313, after adjusting for dispersion. The Pearson’s chi-squared statistic for the Poisson model is 19,239.34, whereas that for the CMP model is 5,812.78, which is a drop of 70%. Adding the dispersion parameter considerably improved the model quality.

**Table 5:** Estimated parameters (standard error), log-likelihood value, Akaike Information Criterion (AIC), and Pearson’s chi-squared value for the Poisson, CMP, ZIP, and ZICMP models.

	Poisson	CMP	ZIP	ZICMP
${\hat{\beta }}_{}$	-1.1974 (0.0128)	-1.1030 (0.0163)	-1.1974 (0.0128)	-1.1030 (0.0163)
$\hat{\delta }$	NA	0.4384 (0.0404)	NA	0.4384 (0.0404)
$\hat{\gamma }$	NA	NA	*-12*.*6700* *(27*.*3600)*	*-14*.*4908* *(42*.*3517)*
Log-Likelihood	-13,872.42	-13,829.48	-13,872.42	-13,829.48
AIC	27,746.84	27,662.97	27,748.84	27,664.97
Pearson’s chi-squared	19,239.34	5,812.78	19,239.29	5,810.13
#Parameters	1	2	2	3

Note: Statistically insignificant coefficients are presented in italics.

The CMP model simply decreases the variability in the Poisson distribution but does not necessarily accommodate excess zeros. The score statistic provided evidence that the observed zeros exceeded the zero bound of the Poisson distribution (177.69, df = 1, p < 0.0001). Interestingly, fitting the zero-inflated models to the data provided a different conclusion. The always-zero proportion parameter in the zero-inflated model was not statistically significant. Neither the ZIP model nor the ZICMP model captured the excess zeros in the data.

The ZI coefficients refer to separating actual non-fatal accidents from crashes with or without deaths. Exponentiating these coefficients in both ZIP (exp(-12.6700) ≈ 0) and ZICMP (exp(-14.4908) ≈ 0) returned an approximate zero value, suggesting that the proportion of actual non-fatal accidents was zero. Thus, observations of zero deaths seem as expected, not excess, in the theorical count distribution. Adding zero-inflation to the model did not improve the log-likelihood or reduce the AIC. Further exploration is required to fully explain the two processes of zeros in the RTA data. In the next section, covariates are added to these models to improve the fit.

### Regression models with covariates

Count regression models were applied to investigate the relationship between the number of deaths and covariates, including road characteristics, weather conditions, light conditions, and month of the year. Before developing the model, the correlations among independent variables were considered using Cramer’s V, which produced values ranging from 0.029 to 0.591. These results showed no significant correlation among covariates and supported the notion that there was no multicollinearity in the regression models.

**Fig 3:**
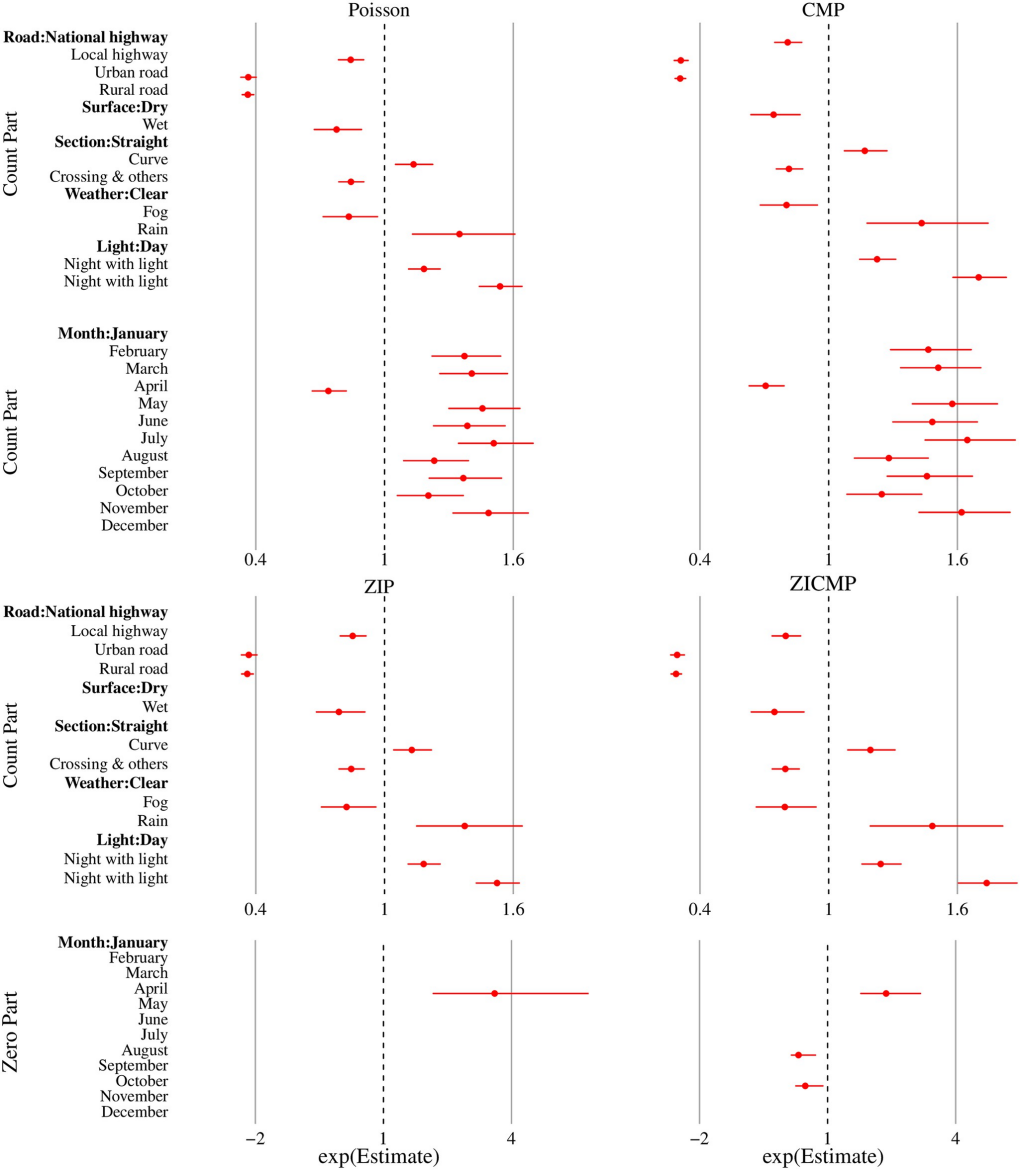
95% confidence interval for statistically significant factors in the Poisson, CMP, and zero-inflated models.

The results of the coefficients and standard errors are listed in Table 6, and the 95% confidence intervals are displayed in Fig 3. Standard Poisson regression and CMP regression were used for the count part modeling with and without an additional dispersion parameter, respectively. Then, the zero-inflated models with ZIP and ZICMP regression were adopted to enhance the model fitting in zero-part modeling.

**Table 6:** Estimated parameters (standard error) from the Poisson, CMP, and zero-inflated regression models. Note that statistically insignificant factors are not presented.

	Poisson	CMP	ZIP	ZICMP
	Count Part		Count Part	
Roadway Class: baseline = National highway				
Rural highway	-0.1715 (0.0356)	-0.2121 (0.0396)	-0.1606 (0.0361)	-0.2236 (0.0423)
Urban Road	-1.0079 (0.0500)	-1.1690 (0.0533)	-1.0013 (0.0504)	-1.2255 (0.0554)
Local street	-1.0136 (0.0378)	-1.1786 (0.0410)	-1.0206 (0.0382)	-1.2427 (0.0431)
Road Surface: baseline = Dry				
Wet	-0.2528 (0.0722)	-0.2969 (0.0785)	-0.2389 (0.0730)	-0.2915 (0.0831)
Road Section: baseline = Straight				
Curve	0.1269 (0.0390)	0.1555 (0.0433)	0.1198 (0.0398)	0.1777 (0.0470)
Crossing and others	-0.1701 (0.0353)	-0.2047 (0.0384)	-0.1686 (0.0356)	-0.2258 (0.0404)
Weather: baseline = Clear				
Fog	-0.1819 (0.0775)	-0.2190 (0.0842)	-0.1947 (0.0784)	-0.2287 (0.0895)
Rain	0.2997 (0.0899)	0.3599 (0.0998)	0.3176 (0.0909)	0.3941 (0.1063)
Light: baseline = Day				
Night with light	0.1688 (0.0317)	0.2034 (0.0350)	0.1676 (0.0322)	0.2170 (0.0374)
Night without light	0.4308 (0.0330)	0.5301 (0.0373)	0.42173 (0.0335)	0.5518 (0.0401)
Month: baseline = January				
	Count Part		Zero Part	
February	0.3168 (0.0593)	0.3814 (0.0653)		
March	0.3413 (0.0571)	0.4126 (0.0629)		
April	-0.3034 (0.0548)	-0.3481 (0.0588)	1.2824 (0.2599)	0.8632 (0.1480)
May	0.3761 (0.0579)	0.4548 (0.0639)		
June	0.3265 (0.0613)	0.3940 (0.0676)		
July	0.4115 (0.0587)	0.4984 (0.0649)		
August	0.2084 (0.0624)	0.2472 (0.0683)		-1.1441 (0.4104)
	Count Part		Zero Part	
September	0.3129 (0.0628)	0.3770 (0.0693)		
October	0.1861 (0.0650)	0.2209 (0.0712)		-0.7514 (0.3205)
November	0.3954 (0.0601)	0.4820 (0.0667)		
December				
$\hat{\beta }$	-1.1002 (0.0427)	-0.9091 (0.0472)	-0.8269 (0.0236)	-0.4400 (0.0326)
$\hat{\gamma }$	NA	NA	-1.7682 (0.2508)	-0.9714 (0.1350)
$\hat{\delta }$	NA	0.7381 (0.0316)	NA	0.8654 (0.0297)
Log-Likelihood	-12950.99	-12785.35	-13017.66	-12784.21
AIC	25946	25616.69	26081.31	25616.42
Pearson’s chi-squared	17980.32	5294.07	17123.84	5260.38
No. of Parameters	22	23	23	24

### Count part in Poisson and CMP regression

According to the log-likelihood, AIC, and Pearson’s chi-squared values, CMP regression outperformed ordinary count regression. All six covariates were statistically significant predictors of the fatality distribution. Driving on national highways or dry surfaces is associated with a higher fatality rate. On average, the number of deaths at crossings is likely to decrease compared with that on straight road sections. However, the opposite is true when approaching curves. The positive light condition coefficient implied that driving during daylight was much safer than driving at night, with or without light on the streets. RAFs were most common on sunny days. A higher risk was observed during rain, but the risk was lower in foggy weather. Compared to the occurrence of RAFs in January, the fatality risk decreased in April, but increased in other months. For each predictor, the 95% confidence intervals estimated from the CMP regression were wider than those from the Poisson regression. This is because of the adjustment for the underdispersion phenomenon.

### Count part and zero part in ZIP and ZICMP regression

The ZICMP model provides a better fit to fatality frequency data than the ZIP model. Five risk factors are statistically significant for predicting the count part distribution, whereas only the month of the year is an important covariate for extracting excessive zeros from the data. The zero-inflated model coefficients separate actual non-fatal accidents from crashes with or without deaths. The actual number of non-fatal accidents was 2.37 (exp(0.8632)) times higher in April than in January. The estimated proportion of the always-zeros was 0.275 and 0.473 (refer to Eq (6)) for the reference months of January and April, respectively. More specifically, the extra zeros were associated with an excessive number of no deaths that can be explained by the month factor.

Using January as a baseline, the probability of zero deaths significantly increases in April, but decreases in August and October. The probability of zero deaths in other months did not differ from that in the first month of the year. These results confirm the exploratory data analysis results that the death rate is lower during festival periods, despite an unusual increase in the number of non-fatal accidents. This information was not explicit when fitting the data with Poisson or CMP regression. For the count part, the directions of associations between the response and the roadway class, surface, and section as well as lighting and weather conditions, were the same as those presented in the CMP regression result. However, the magnitudes were clearly different because the ZICMP model allows for variable adjustment in underdispersed data along with zero-inflation. The corresponding confidence intervals were estimated to reflect both phenomena.

When controlling for covariates, the expected number of deaths per accident increased from 0.333 under the Poisson regression to 0.393 under the ZICMP regression. In other words, after adjusting for underdispersion and zero-inflation, for every ten accidents, the number of human deaths approximately increased from three to four people. This finding is of vital importance for public health concerns and road safety issues in Thailand.

## Discussion

### Model selection

The Poisson model is the most widely used model for analyzing count data. However, the Poisson model has the drawback of assuming a unit variance-to-mean ratio. In road safety data, this restriction may be violated because the observed count may present a variance that is larger or smaller than expected, resulting in overdispersion or underdispersion, respectively. NB regression is often used to model RTAs, RAFs, or RAIs with overdispersion, such as, the number of injury accidents on road bridges in Norway during 2010–2016 [59]; RAFs and RAIs with spatial panel data analysis in Thailand during 2012–2016 [60]; the number of human deaths per crash in the Oromia region of Ethiopia [61]; and safety performance functions for urban intersections of Antwerp in Belgium [62].

CMP regression is an alternative model to NB regression and has the advantage of being able to handle both overdispersed and underdispersed counts. However, CMP is not popular because of the complexity in estimating the mean and dispersion parameters using iterative methods for nonlinear optimization and is limited by the availability of computational tools. A generalized linear model based on CMP distribution was developed by Guikema et al. [63] and was first applied to the analysis of RTA data with overdispersion [28]. Two studies of crash data were from signalized four-legged intersections in Toronto, Ontario in 1995, and from rural four-lane divided and undivided highways in Texas. The results of the goodness-of-fit test statistic and predictive performance were similar for the CMP and NB regressions.

Modeling RTAs, RAFs, or RAIs with underdispersed data has rarely been reported. A study in 2010 [30] showed that the CMP model for underdispersion provides a better fit to RTAs than the Poisson and gamma models in an analysis of 162 railway-highway crossings in South Korea between 1998 and 2002. Further, a recent report in 2020 introduced clustered longitudinal CMP with gamma random effects to model RTAs in Mauritius [29].

Excessive zero counts are highly skewed to the right and may cause a smaller conditional mean than the true mean value, resulting in overdispersion or underdispersion. The zero-inflated version models of Poisson, NB, and CMP can be applied to overcome such problems. For example, Poisson, NB, ZIP, and ZINB were used to model the number of RAFs on the roads with the highest number of accidents in Malaysia, F0050 [64], and in the Oromia region of Ethiopia [61]. Modeling the zero-inflated regression of Poisson and NB of the number of RTAs on roads such as the F001 Jalan Jb Air Hitam and FT050 Kluang–A/Hitam–B/Pahat roads in Malaysia was reported in [65] and [66], respectively. The results suggest that the zero-inflated version models offer better statistical performance than traditional models.

Recently, the ZICMP distribution and regression, along with their computational “COMPoissonReg” package in R, were developed [52]. ZICMP can account for excess zeros and data with overdispersion or underdispersion. The model performance was compared to that of ZIP and ZINB in a study of educational data with overdispersion. As expected, ZICMP and ZINB exhibited similar performances in capturing extra zeros, overdispersion, and surpassing ZIP. To the best of our knowledge, this has not yet been used for road safety analysis.

In this study, the Poisson, CMP, ZIP, and ZICMP regression models were fit to RAFs that occurred in Thailand in 2015. This is an interesting area because excess zeros and underdispersion are rarely observed in road safety data. The ZICMP model performed the best, marginally outperforming CMP with a minute, almost negligible, difference in log-likelihood values. This suggests that having many zeros does not necessarily mean a zero-inflated model is required or that important covariates are missing. Therefore, issues of concern that need to be addressed, and the possibility of finding associated factors dictate the model-selection process. These two distinct aspects are clear. CMP provides insight into death counts per crash if there are no extra zeros. When an accident occurs, the driver or passenger may be injured or die. ZICMP accounts for excessive zeros, suggesting an unusual increase in the number of zero death counts on roads. This can be viewed as unexpected, non-fatal accidents. The extraction of such information requires the identification of associated covariates.

### Month, road, and environmental effects

The month of the year played a vital role in extracting different messages from the underdispersed fatality data. In the case of death counts, the count part in CMP addresses the lower number of fatalities in April and the larger number of deaths in other months compared to January. For the extra zeros, the zero part in ZICMP captures excess zero deaths in April, but figures are lower in August and October than in January. However, the month did not affect the death counts in the count part of the ZICMP model. This indicates that different sets of variables are required to interpret different aspects of the data. Our results serve as the basis of the model formulation. These two generalized linear mixed models allow for different sets of relationships between the average, dispersion, or zero structure and its associated covariates [52].

Two key findings were highlighted in April: a lower number of deaths and a greater number of excess zero deaths on roads. This is attributed to April being a festival month. For centuries, April has been celebrated annually as the traditional Thai New Year, known as the Songkran festival (the period of 11–17 April every year). It is rich in culture and tradition with water blessing ceremonies for prosperity and wellness as well as the world-renowned water parties for ritual cleansing and a fresh start. An unacceptable drawback is that it coincides with the time of the highest crash frequencies of the year, called the ‘‘seven deadly days.”

Heavy road traffic occurs at the beginning and end of the long holidays when there is an excess of public transportation and numerous private vehicles on the roadways connecting the provinces. Because rail public transit and railroad networks in Thailand are not efficiently served, public buses must double or even triple the number of services to accommodate the most prominent human migration of the year. The traffic flow rate on the road is low, which causes traffic jams and accidents. Road accidents may cause more congestion, particularly on interconnected and national highways.

For decades, the government accorded a lot of time, budget, and effort into road safety campaigns to raise awareness and change attitudes and behaviors, particularly during festivals. However, thus far, all earlier campaigns have shown no reduction in fatalities [9,11–13]. A new insight from our study shows that the number of deaths is not very different between the months, but the number of non-fatal accidents is. Interestingly, our findings contradict Thai people’s risk beliefs and public awareness of RAFs. Advertising campaigns emphasize death counts and do not mention accident counts, claiming that the number of deaths on the roads is dramatically higher than usual during festivals. This is deceptive. Accidents related to injuries and property damage are also harmful to the social and economic aspects of society. Disability after RAIs is a massive health problem and the economic consequences have resulted in a loss of gross domestic product in the country by 3%–6% or 273–545 million Baht (US$ 8.3–16.5 million, based on an exchange rate of 33 Baht to US$ 1) [60]. Therefore, the authorities are recommended to implement adequate road safety measures for road users daily, not just for a specific period.

Most studies in the literature concur that the factors affecting RTAs and RAFs are roads and the environment. The present study yielded comparable results. The correlations between traffic accidents and road conditions, environmental factors and population were significant [67]. The female sex, the presence of alcohol, and a high degree of curvature in the road all increase the likelihood of severe traffic accidents [68]. Human behavior and demographics are also known factors impacting road safety [69] and thus may be considered in further studies in Thailand.

## Conclusion

In this study, the CMP regression and its zero-inflation version, ZICMP regression, were considered for an underdispersion problem and excessive zero death counts for RAFs in Thailand. Neither Poisson regression nor ZIP regression is appropriate for modeling this type of count response. Because underdispersion is not accounted for, the classic models produce a lower standard error of the estimated parameters than expected. This study highlights the fact that having many zeros does not necessarily mean that a zero-inflated model is required; it depends on the issues of concern that need to be addressed. Finding associated factors to capture excessive zeros is challenging but can provide insights into what happened. A key finding is that the month of the year has the power to determine the difference between the death counts and the extra zero death counts, referred to as uncommon non-fatal accidents in this study. Using January as a baseline, the ZICMP regression revealed that the largest number of non-fatal accidents were in April when people celebrated the traditional Thai New Year Songkran festival. This figure was not obtained using the Poisson or CMP regression. The CMP regression shows a lower fatality rate during this festival month because the number of deaths did not change much throughout the year, but the number of accidents was considerably higher in April than in other months. Other covariates, including road (class, surface, and section) and environment (weather and light conditions), were significant predictors of RAFs in Thailand, as commonly reported elsewhere.

Underdispersion problems in road accident data are rare; however, they have been reported in relation to RAFs in Thailand. In addition to the CMP model, the geometric model may serve as an alternative to the familiar Poisson model. The advantage of the geometric distribution is that it is a mixture of Poisson and an exponential distribution; thus, it incorporates some form of heterogeneity. This point is left for future research. In addition, human behavior, vehicles, roads, and the environment interact with each other during accidents. A possible enhancement of road safety databases, in which various authorities are involved in supplying such data, is a key to effective road safety measures, to reduce road accidents, injuries, and fatalities, not only in Thailand but worldwide.

## Supporting information

S1 Table Codes of the independent variables. Each categorical covariate was divided into different levels. Different levels were coded in different values.
(DOCX)

S1 File. Raw data Data specific to this study is provided. The number of deaths per accident in 2015 ranged from 0 to 6, with many accidents reporting 0 or 1 death per crash.
(CSV)

S2 File Data for visualizing in Fig 1 is provided. A trend of the number of deaths per accident and death rate per 100,000 people is illustrated over the last ten years, 2011–2020. Two reports of death counts during Songkran festival and New Year holiday are given by the Injury Data Collaboration Center (IDCC) and Department of Disaster Prevention and Mitigation (DDPM).
(XLSX)

S3 File Data for visualizing in Fig 2 is provided. The number of fatal accidents was not quite different over the year, but the number of non-fatal accidents was higher in festival months (January, April, and December). The death rate in these festival months was lower than other months of the year.
(XLSX)

S4 File The 95% confidence intervals for the coefficients from count regression models in Fig 3 are provided. The month of the year was the statistically significant predictor of human death according to the ZIP and ZICMP regression.
(XLSX)

## References

[1] World Health Organization. Global status report on road safety 2015. p. 323.

[2] Global Status Report on Road Safety 2018. 10.1136/ip.2009.023697 19652008

[3] Klungboonkrong P, Faiboun N, Woolley J. Modelling road accident fatalities in Thailand and other Asian countries. Int J Geomate. 2018;15(52):91–98.

[4] Global Road Safety Performance Targets [Internet]. 2020. http://apps.who.int/bookorders.

[5] World Health Organization. Global Plan for Decade of Action for Road Safety 2021–2030. 2021. https://www.who.int/teams/social-determinants-of-health/safety-and-mobility/decade-of-action-for-road-safety-2021-2030.

[6] Division of Injury Prevention, Department of Disease Control, Ministry of Public Health, Thailand Injury Data Collaboration Center. 2021. https://dip.ddc.moph.go.th/new/.

[7] Hordofa GG, Assegid S, Girma A, Weldemarium TD. Prevalence of fatality and associated factors of road traffic accidents among victims reported to Burayu town police stations, between 2010 and 2015, Ethiopia. J Transp Heal. 2018;10:186–193.

[8] Department of Disaster Prevention and Mitigation, Ministry of Interior, Thailand. Office of General Secretariat of Road Safety Operation Center. 2021. http://roadsafety.disaster.go.th/.

[9] Tanaboriboon Y, Satiennam T. Traffic accident in Thailand. IATSS Res. 2005;29(1):88–100.

[10] Chadbunchachai W, Suphanchaimaj W, Settasatien A, Jinwong T, Kaen Regional Hospital K, Kaen K. Road traffic injuries in Thailand: Current situation [Internet]. J Med Assoc Thai. 2012;95:S274–S281. Available from: http://jmat.mat.or.th. 23130465

[11] Siviroj P, Peltzer K, Pengpid S, Morarit S. Helmet use and associated factors among Thai motorcyclists during Songkran festival. Int J Environ Res Public Health. 2012;9(9):3286–3297. doi: 10.3390/ijerph9093286 23202686 PMC3499868

[12] Siviroj P, Peltzer K, Pengpid S, Morarit S. Non-seatbelt use and associated factors among Thai drivers during Songkran festival. BMC Public Health. 2012;12(1):1–7. doi: 10.1186/1471-2458-12-608 22863275 PMC3490791

[13] Riyapan S, Thitichai P, Chaisirin W, Nakornchai T, Chakorn T. Outcomes of emergency medical service usage in severe road traffic injury during Thai holidays. West J Emerg Med. 2018;19(2):266–275. doi: 10.5811/westjem.2017.11.35169 29560053 PMC5851498

[14] Sangkharat K, Thornes JE, Wachiradilok P, Pope FD. Determination of the impact of rainfall on road accidents in Thailand. Heliyon. 2021;7(2):e06061. doi: 10.1016/j.heliyon.2021.e06061 33644437 PMC7895724

[15] Champahom T, Jomnonkwao S, Banyong C, Nambulee W, Karoonsoontawong A, Ratanavaraha V. Analysis of crash frequency and crash severity in Thailand: Hierarchical structure models approach. Sustain. 2021;13(18):10086.

[16] Leardsuwansri R, Phonsrirat C, Prawalwanna P, Wongsai N, Wongsai S, Simmachan T. Road traffic injuries in Thailand and their associated factors using Conway-Maxwell-Poisson regression model. Thai J. Math. 2022;240–249.

[17] Taveekal P, Rajchanuwong P, Wongwiangjan R, Lerdsuwansri R, Intrakul J, Simmachan T, et al. Modelling road accidents injuries and fatalities in Suratthani province of Thailand using Conway-Maxwell-Poisson regression. Thailand Stat. 2023. (in press).

[18] Michener R, Tighe C. A Poisson regression model of highway fatalities. Am. Econ. Assoc. 1992;82(8):452–456.

[19] Hezaveh AM, Arvin R, Cherry CR. A geographically weighted regression to estimate the comprehensive cost of traffic crashes at a zonal level. Accid Anal Prev. 2019;131:15–24. 31233992 10.1016/j.aap.2019.05.028

[20] Chen T, Sze NN, Chen S, Labi S. Urban road space allocation incorporating the safety and construction cost impacts of lane and footpath widths. J Safety Res. 2020;75:222–232. doi: 10.1016/j.jsr.2020.09.014 33334480

[21] Obinguar DD, Iryo-Asano M. Macroscopic analysis on the frequency and severity of pedestrian crashes on National Roads in Metro Manila, Philippines. IATSS Res. 2021;45(4):521–529.

[22] Das DK. Exploring the significance of road and traffic factors on traffic crashes in a South African city. Int J Transp Sci Technol. 2022.

[23] Quistberg DA, Hessel P, Rodriguez DA, Sarmiento OL, Bilal U, Caiaffa WT, et al. Urban landscape and street-design factors associated with road-traffic mortality in Latin America between 2010 and 2016 (SALURBAL): An ecological study. Lancet Planet Heal. 2022;6(2):e122–e131. doi: 10.1016/S2542-5196(21)00323-5 35150622 PMC8850369

[24] Khattak MW, Pirdavani A, De Winne P, Brijs T, De Backer H. Estimation of safety performance functions for urban intersections using various functional forms of the negative binomial regression model and a generalized Poisson regression model. Accid Anal Prev. 2021;151. 33421730 10.1016/j.aap.2020.105964

[25] Kamla J, Parry T, Dawson A. Analysing truck harsh braking incidents to study roundabout accident risk. Accid Anal Prev. 2019;122:365–377. doi: 10.1016/j.aap.2018.04.031 29739619

[26] Safaei N, Zhou C, Safaei B, Masoud A. Gasoline prices and their relationship to the number of fatal crashes on U.S. roads. Transp Eng. 2021;4:100053.

[27] Cai M, Yazdi MAA, Mehdizadeh A, Hu Q, Vinel A, Davis K, et al. The association between crashes and safety-critical events: Synthesized evidence from crash reports and naturalistic driving data among commercial truck drivers. Transp Res Part C Emerg Technol. 2021;126:103016.

[28] Lord D, Guikema SD, Geedipally SR. Application of the Conway-Maxwell-Poisson generalized linear model for analyzing motor vehicle crashes. Accid Anal Prev. 2008;40(3). doi: 10.1016/j.aap.2007.12.003 18460381

[29] Khan NM, Soobhug AD, Jannoo Z. Modeling road traffic accidents in Mauritius using clustered longitudinal COM-Poisson with gamma random effects. Commun Stat Case Stud Data Anal Appl. 2020;7(2):113–127.

[30] Lord D, Geedipally SR, Guikema SD. Extension of the application of Conway-Maxwell-Poisson models: Analyzing traffic crash data exhibiting underdispersion. Risk Anal. 2010;30(8):1268–1276. doi: 10.1111/j.1539-6924.2010.01417.x 20412518

[31] Mansfield TJ, Peck D, Morgan D, McCann B, Teicher P. The effects of roadway and built environment characteristics on pedestrian fatality risk: A national assessment at the neighborhood scale. Accid Anal Prev. 2018;121:166–176. doi: 10.1016/j.aap.2018.06.018 30248532

[32] Raihan MA, Alluri P, Wu W, Gan A. Estimation of bicycle crash modification factors (CMFs) on urban facilities using zero inflated negative binomial models. Accid Anal Prev. 2019;123. doi: 10.1016/j.aap.2018.12.009 30562669

[33] Wan Y, Li Y, Liu C, Li Z. Is traffic accident related to air pollution? A case report from an island of Taihu Lake, China. Atmos Pollut Res. 2020;11(5):1028–1033.

[34] Mathew J, Benekohal RF. Highway-rail grade crossings accident prediction using zero inflated negative binomial and empirical Bayes method. J Safety Res. 2021;79:211–236. doi: 10.1016/j.jsr.2021.09.003 34848003

[35] Cloutier MS, Lachapelle U. The effect of speed reductions on collisions: A controlled before-and-after study in Quebec, Canada. J Transp Heal. 2021;22:101137.

[36] Hordofa GG, Assegid S, Girma A, Weldemarium TD. Prevalence of fatality and associated factors of road traffic accidents among victims reported to Burayu town police stations, between 2010 and 2015, Ethiopia. J Transp Heal. 2018;10:186–193.

[37] Casado-Sanz N, Guirao B, Gálvez-Pérez D. Population ageing and rural road accidents: Analysis of accident severity in traffic crashes with older pedestrians on Spanish crosstown roads. Res Transp Bus Manag. 2019;30.

[38] Nguyen DVM, Vu AT, Polders E, Ross V, Brijs T, Wets G, et al. Modeling the injury severity of small-displacement motorcycle crashes in Hanoi City, Vietnam. Saf Sci. 2021;142:105371.

[39] Singh G, Sachdeva SN, Pal M. M5 model tree based predictive modeling of road accidents on non-urban sections of highways in India. Accid Anal Prev. 2016;96:108–117. doi: 10.1016/j.aap.2016.08.004 27521904

[40] Chen MM, Chen MC. Modeling road accident severity with comparisons of logistic regression, decision tree and random forest. Inf. 2020;11(5).

[41] Fiorentini N, Losa M. Handling imbalanced data in road crash severity prediction by machine learning algorithms. Infrastructures. 2020;5(7).

[42] Philip AO, Saravanaguru RAK. Secure incident & evidence management framework (SIEMF) for internet of vehicles using deep learning and blockchain. Open Comput Sci. 2020;10(1):408–421.

[43] Nour MK, Naseer A, Alkazemi B, Jamil MA. Road traffic accidents injury data analytics. Int J Adv Comput Sci Appl. 2020;11. Available from: www.ijacsa.thesai.org.

[44] Meocci M, Branzi V, Martini G, Arrighi R, Petrizzo I. A predictive pedestrian crash model based on artificial intelligence techniques. 2021. doi: 10.3390/app112311364

[45] Guo Y, Sayed T, Essa M. Real-time conflict-based Bayesian Tobit models for safety evaluation of signalized intersections. Accid Anal Prev. 2020;144. doi: 10.1016/j.aap.2020.105660 32623321

[46] Guo Y, Sayed T, Zheng L. A hierarchical Bayesian peak over threshold approach for conflict-based before-after safety evaluation of leading pedestrian intervals. Accid Anal Prev. 2020;147. doi: 10.1016/j.aap.2020.105772 32949863

[47] Guo Y, Li Z, Liu P, Wu Y. Modeling correlation and heterogeneity in crash rates by collision types using full Bayesian random parameters multivariate Tobit model. Accid Anal Prev. 2019;128. doi: 10.1016/j.aap.2019.04.013 31048116

[48] Guo Y, Osama A, Sayed T. A cross-comparison of different techniques for modeling macro-level cyclist crashes. Accid Anal Prev. 2018;113. doi: 10.1016/j.aap.2018.01.015 29407667

[49] Conway RW, Maxwell WL. Network dispatching by the shortest-operation discipline. Oper Res. 1962;10(1):51–73.

[50] Shmueli G, Minka TP, Kadane JB, Borle S, Boatwright P. A useful distribution for fitting discrete data: Revival of the Conway-Maxwell-Poisson distribution. J R Stat Soc Ser C Appl Stat. 2005;54(1):127–142.

[51] Sellers KF, Shmueli G. A flexible regression model for count data. Ann Appl Stat. 2010;4(2):943–961.

[52] Sellers KF, Raim A. A flexible zero-inflated model to address data dispersion. Comput Stat Data Anal. 2016;99:68–80.

[53] van den Broek J. A score test for zero inflation in a Poisson distribution. Biometrics. 1995;51(2):738–743. 7662855

[54] R Core Team. R A Lang Environ Stat Comput R Found Stat Comput Vienna, Austria URL http://www.R-project.org. 2021.

[55] Venables WN, Springer BDR. Modern applied statistics with S. 4th ed. Springer New York, NY; 2002. http://www.insightful.com.

[56] Zeileis A, Kleiber C, Jackman S. Regression models for count data in R. J Stat Softw. 2008;27(8):1–25.

[57] Sellers KF, Lotze T, Raim A. Package COMPoissonReg. Package “COMPoissonReg.” 2019.

[58] Friendly M. “vcd” and “vcdExtra” packages in R. CRAN Repository. 2017;171.

[59] Elvik R, Sagberg F, Langeland PA. An analysis of factors influencing accidents on road bridges in Norway. Accid Anal Prev. 2019;129:1–6. doi: 10.1016/j.aap.2019.05.002 31078947

[60] Suphanchaimat R, Sornsrivichai V, Limwattananon S, Thammawijaya P. Economic development and road traffic injuries and fatalities in Thailand: An application of spatial panel data analysis, 2012–2016. BMC Public Health. 2019;19(1):1–15. 31684951 10.1186/s12889-019-7809-7 PMC6829991

[61] Aga MA, Woldeamanuel BT, Tadesse M. Statistical modeling of numbers of human deaths per road traffic accident in the Oromia region, Ethiopia. PLoS One. 2021;16(5):e0251492. doi: 10.1371/journal.pone.0251492 34010290 PMC8133474

[62] Khattak MW, Pirdavani A, De Winne P, Brijs T, De Backer H. Estimation of safety performance functions for urban intersections using various functional forms of the negative binomial regression model and a generalized Poisson regression model. Accid Anal Prev. 2021;151:105964. doi: 10.1016/j.aap.2020.105964 33421730

[63] Guikema SD, Coffelt JP. Modeling count data in risk analysis and reliability engineering. In: Handbook of Performability Engineering. 2008.

[64] Musa WZ, Prasetijo J, Zainal ZF. Road fatality model based on over-dispersion data along Federal Route F0050. In: MATEC Web of Conferences. 2017.

[65] Prasetijo J, Musa WZ. Modeling zero-Inflated regression of road accidents at Johor Federal Road F001. In: MATEC Web of Conferences. 2016.

[66] Prasetijo J, Musa WZ, Mohd Jawi Z, Zainal ZF, Hamid NB, Subramaniyan A, et al. Vehicle road accident prediction model along federal road FT050 Kluang-A/Hitam-B/Pahat route using excess zero data. In: IOP Conference Series: Materials Science and Engineering. IOP Publishing Ltd; 2020.

[67] Hashimoto S, Yoshiki S, Saeki R, Mimura Y, Ando R, Nanba S. Development and application of traffic accident density estimation models using kernel density estimation. J Traffic Transp Eng (English Ed.) 2016;3(3):262–270.

[68] Schneider WH, Savolainen PT. Comparison of severity of motorcyclist injury by crash types. Transp Res Rec. 2011;2265:70–80.

[69] Adanu EK, Hainen A, Jones S. Latent class analysis of factors that influence weekday and weekend single-vehicle crash severities. Accid Anal Prev. 2018;113:187–192. doi: 10.1016/j.aap.2018.01.035 29426023

