# Correction: Modeling road accident fatalities with underdispersion and zero-inflated counts

**DOI:** 10.1371/journal.pone.0309234

**Published:** 2024-08-15

**Authors:** Teerawat Simmachan, Noppachai Wongsai, Sangdao Wongsai, Rattana Lerdsuwansri

The funding statement for this paper is incorrect. The correct funding statement is: This study was supported by the Thammasat Postdoctoral Fellowship (grant number TUPD7/2564; https://research.tu.ac.th/tu-research-funds) and the Thailand Science Research and Innovation Fundamental Fund for the fiscal year 2023. The funders had no role in study design, data collection and analysis, decision to publish, or preparation of the article.
