# Supplementary material for: Modeling road accident fatalities with underdispersion and zero-inflated counts
Source: PLoS One. 2022 Nov 17;17(11):e0269022. doi: 10.1371/journal.pone.0269022 (PMC9671366; doi:10.1371/journal.pone.0269022)
Supplement: S1 Table — Each categorical covariate was divided into different levels. Different levels were coded in different values. (DOCX) [file pone.0269022.s001.docx]

**S1 Table. Codes of the independent variables.**

| Variable | Description | Code |  |
| --- | --- | --- | --- |
| Roadway Class |  |  |  |
|  | National highway | 1 |  |
|  | Rural highway | 2 |  |
|  | Urban road | 3 |  |
|  | Local street | 4 |  |
| Road surface |  |  |  |
|  | Dry | 1 |  |
|  | Wet | 2 |  |
| Road section |  |  |  |
|  | Straight | 1 |  |
|  | Curve | 2 |  |
|  | Crossing and others | 3 |  |
| Weather condition |  |  |  |
|  | Clear | 1 |  |
|  | Fog | 2 |  |
|  | Rain | 3 |  |
| Light condition |  |  |  |
|  | Day | 1 |  |
|  | Night with light | 2 |  |
|  | Night without light | 2 |  |
| Month |  |  |  |
|  | January | 1 |  |
|  | February | 2 |  |
|  | March | 3 |  |
|  | April | 4 |  |
|  | May | 5 |  |
|  | June | 6 |  |
|  | July | 7 |  |
|  | August | 8 |  |
|  | September | 9 |  |
|  | October | 10 |  |
|  | November | 11 |  |
|  | December | 12 |  |
